# The Influence of Culture on Professional Skill

**Published:** 1887-03

**Authors:** Thomas Fillebrown


### Independent Practitioner.

VOL. VIII. MARCH, 1887. NO. 3.

### Original Communications.

NOTE.—No paper published or to be published in another journal will be accepted for this department. All papers must be in the hands of the Editor before the first day of the month preceding that in which they are expected to appear. Extra copies will be furnished to each contributor of an accepted original article, and reprints, in pamphlet form, may be had at the cost of the paper, press-work and binding, if ordered when the manuscript is forwarded. The Editor and Publishers are not responsible for the opinions expressed by contributors. The journal is issued promptly, on the first day of each month.

### THE INFLUENCE OF CULTURE ON PROFESSIONAL SKILL.

ANNUAL ADDRESS BEFORE THE MASSACHUSETTS DENTAL SOCIETY, AT BOSTON, DECEMBER 9, 1886.

BY THOMAS FILLEBROWN, M. D., D. M. D.

Ever since Arachne tried her skill with the gods and brought down the wrath of Minerva for her temerity, manipulative skill has been the active means of the world's material progress. While agriculture has always been and is the source of the world's greatest wealth, and furnishes the essentials of the people's support, handiwork has ever ministered in so many ways to personal comfort, has created so much of wealth and aided social progress to such an extent as to give it the first rank as a civilizing force. Skill clothes the person, shelters from storm, defends against the strong, creates machinery and sustains its busy hum, delves in the earth and brings forth its treasures and converts them into forms for comfort or ornament, or into forms of power to better subserve the wants of the working and waiting millions on the earth. It furnishes the means of interchange of the productions of different parts of the earth. It makes it possible to send manufactured commodities to distant regions, and maintains social intercourse between all parts of the world. It executes works of art to please the eye, and constructs articles of vertu to satisfy the taste.

Emerson says, "There is no division in art as between fine art and practical art." Whether art be subject to such division or not, there is a very radical difference in the products. On the one hand, we have the conception of the imagination exhibited in sculpture and painting, conceived by the brain and executed by the hand of the artist; on the other, the practical results shown in the mill, the forge, the steamship, the railway and telegraph. For our present purpose we need not discuss such division. At the present time we deal with the skill which makes the construction of both possible, and seek to find how such skill has been influenced for better or worse by culture of the mind..

Culture and manipulative skill have ever been considered as something apart from each other, and until comparatively recent years were considered almost, if not quite, antagonistic. The consideration of culture, as such, is of recent origin and of slow growth, yet it has now gained ascendency in the minds of the thinking and ruling classes, and firmly holds its sway.

As no man is wholly without skill nor entirely without culture, it is difficult to properly estimate in him the influence the one has on the other. Constructive skill can exist without culture or mental ability, as is shown in the web of the spider, the honeycomb of the bee, the nest of the bird and the cocoon of the silk-worm. Skill alone has no originating power; it does the same thing in the same way, time after time, through endless repetitions.

The very names of the ages show the intimate connection of the useful arts with the progress of civilization, and mark the epochs of the world's progress in culture as well as art. In the crude age of stone, men were savages; they had no accumulation of property, no division of labor, no social organization, no written laws, no tools better than stone. The next era was marked by the discovery of bronze, and the world emerged from a state of savagery into the enlightenment of barbarism. Bronze afforded better weapons for war, and made cultivation of the soil possible, and people began to have a locality and some attachment to a home. Barbarism discovered the art of quarrying and cutting stone, of spinning and weaving, of brick-making, and left to the world as its monuments the Pyramids of Egypt, the Temple of Karnak, the Sphinx and the Obelisks. The iron age produced the alphabet and a refined taste in literature and art. As the individual begins his existence in the utmost helplessness, ignorance and speechlessness, and through long struggles of years becomes the man, so the race struggled through ages of savage ignorance and barbarism before it could be entitled to the name of a people.

Until very recently, manipulative skill was considered menial, and was not practiced by any person who presumed to any mental or social attainments. Plato had no place for the artisan in his republic, and Socrates held him in contempt. The artisan of the middle ages was fit only to be a slave, and lived and died as such. The wonderfully fine work of Egyptian temples and Roman monuments was the handiwork of slaves, directed by the cultured brains of the priesthood. The composition of society at that time placed all knowledge and culture in the priests. The activity of the people was in the army. The plodding, working masses were slaves, and to this class was left the menial work of the artisan. At the present time, though the artisan is freed from slavery, yet throughout the world, especially the old, he is held to belong to a separate class, and to work is not considered the part of a gentleman. If an artisan rises to any considerable social distinction he is forced to ignore, in a large measure, his former position, or at least the practice of his calling. Manipulative skill, when needed to render personal service in a professional way, as in case of the physician and the surgeon—and also our own calling—has always been held in higher esteem, and been exempt from the odium attached to ordinary skill exercised on inanimate objects, and it is to such as these our inquiry is especially directed.

The progress of mental culture seems to be divided into three distinct periods—the savage, the barbarous or mediæval, and the modern or age of civilization. During the first—called also the stone age—mental culture made hardly a beginning. Force was the only law known or acknowledged, and the best man physically was the best among his people. Saul was admired for his physical perfection quite as much as for his mental qualities. The middle ages showed great progress of the race. Agriculture was raised to a system and became an honorable calling. Implements of bronze, and later of iron, made improvement in material warfare possible, and made it practicable for the higher conceptions of cultured minds to be worked out in the ornamental buildings of this age. During this period the alphabet came into use, and a refined taste in literature and art was developed; and here the connected story of culture-history begins. During the modern age culture has made rapid and great advances, and become widely disseminated, and the common people feel its influence in a great degree.

The term "culture" is used in quite a variety of senses. Its primitive sense, as applied to the mind, is to improve, enlarge and perfect any single quality, talent or taste without reference to other powers. Applied in this way, it makes the man of one idea. One says: "Industrial art is the main force of culture," thus making culture an impossiblity without art, and secondary to it. A prevalent idea is that knowledge of facts and things is culture. The truer idea is that knowledge makes culture possible. Facts are not science, but their classification makes science.

Matthew Arnold gives the æsthetic conception of culture. He says: "It is a pursuit of our total perfections by means of getting to know, on all matters which most concern us, the best which has been thought and said in the world." Again he says: "Culture is the study of perfection, and leads us to conceive of true human perfection as an harmonious perfection developing all sides of our humanity, and as a general perfection developing all parts of our society." He would have a cultured class, to which he professes to belong, whose aim, calling and end is culture, whose hands are not soiled by toil, whose minds are not disturbed by the disputations of the day or ruffled by interest in the vital questions of the hour. Home rule has no need of them; war or peace is a matter of secondary importance, and they have no hand to raise for or against it. Tariff, free trade, revenue and taxes are beneath their notice, and whether man shall be bond or free, ignorant or educated, rude or cultivated, is to them only an idea. For the working-out of the many social problems, they have no practical suggestions to make.

Such a culture can hardly fail to lead to feelings so æsthetic as to lose all sight of reason or sense, and become only an unworthy dilettanteism, and lead one to become what culture so entirely condemns

—a man of one idea. While the tendency of such culture is to draw apart from the world, the result, as conceived by Mr. Arnold, is far better. He says further: "Culture, which simply means trying to perfect one's self and one's mind as part of one's self, brings us the knowledge that the really blessed thing is to like what right reason ordains, and to follow her authority." This is but another statement of the "law of liberty;" a liberty always to do as one pleases, because one always chooses to do just the right thing, and only that.

M. Renan offers a sound reason for the existence of a cultured class: "The sound instruction of the people is an effect of the high culture of certain classes. Countries which have created a considerable popular instruction, without any serious, higher instruction, will long have to expiate this fault by their intellectual mediocrity, their vulgarity of manners, their superficial spirit, their lack of general intelligence."

While it cannot be denied that a cultured class, even as a special class, may do a work others cannot, the practical American mind appreciates more fully the cultured mind dealing with practical things. It sympathizes with the practical definition of a cultured man, which Mr. Arnold has given in his riper years, in entering the domain of politics and clashing swords with the old gladiator Gladstone, and seeking to make the power of a cultured mind felt on the questions of the day.

Emerson's conception of culture seems to be nearer the true idea, nearer the needs of the human mind, and leaves not out the affections and passions of the human heart. Says he: "Culture is the suggestion from certain best thoughts that a man has a range of affinities through which he can modulate the violence of any master-tones that exert a droning preponderance in his scale." A definition worthy the subject and worthy the man. It makes culture not the end and aim, but the means by which a man makes the most of himself for other and better purposes, and better fits him for his work in life; not of unfitting him for it and withdrawing him from action and joining him to a separate class.

Culture redresses a man's balance and puts him at ease among his equals and superiors; it kills his exaggeration and conceit; it opens his sense of beauty. Culture enlarges the mind and increases its power of conception, both ideal and practical. Again I quote

Emerson: "Culture corrects the theory of success. A topical memory makes a man an almanac; a talent for debate, a disputant; skill to get money makes him a miser. Culture reduces these inflammations by invoking the aid of other powers against the dominant talent."

The pest of society is the egotist. There are dull and bright, sacred and profane, coarse and fine egotists. "'Tis a disease that, like influenza, falls on all constitutions. This egotism has its root in the cardinal necessity by which each individual persists to be what he is. This individuality is not only not inconsistent with culture, but is the basis of it. He only is a well-made man who has a good determination. The end of culture is not to destroy this, but to train away all impedimenta and mixture, and leave nothing but pure power." A man lives a beggarly life who lives only to be practically useful—it is not enough to simply be the block to scotch the wheel; such a one knows little of the possibilities of life.

An early substantial education is the true basis of culture, "though some seem to culture born." If possible every one "should have such opportunities for education and culture that they shall not feel at thirty or forty years that this which I might do is made hopeless through my want of weapons." Culture interests the man more in his public than in his private qualities, and makes a cheerful, intelligent face the index of a cheerful, disciplined mind. Culture enlarges the heart, makes one more polite, more just, more forbearing, more forgiving. Talents, knowledge, skill, are the uncut diamond; culture is the lapidary which grinds the rough into form and perfects the surface and angles which disclose its wondrous beauties and power. There are times when culture must be lost to sight, and what culture has done must be depended upon; times when action is called for, and the why it should be done, and what the result will be, cannot be considered; times when theory is of no avail and only action will serve. Gen. Weitzell, one of the most accomplished and best read men in the army, said to one of his officers, during the late war: "Do not stop to study theory in active campaign—now is the time to be governed by what you see, and not by what you read."

The daily duty of the professional man ought not to be burdened with the conscious thought of the literature and culture of his calling. This must be dismissed, and skill, steadiness and judgment, which are the results of previous training, must alone be depended upon. Anything less than this proves the want of the culture which is so much desired. Specialism without culture is limiting in its influence, dwarfing the intellect, shrivelling the social qualities, and lessening the influence of every such one among his fellow-men. It makes a man more selfish, more conceited and opinionated; his world revolves on a point, is suspended by a hair and his horizon is limited to a microcosm. No man is born with a mind large enough, or strong enough, or with heart warm enough to stand against its influence.

A notable example of this class was the late Horace Greeley. Born with a mind which immediately grasped the problems of life and the living questions of the day, it jumped the ordinary course of training and spurned the slow process of mental discipline, and grasped by reading what generally is acquired by hard study. Politics was to him the end and aim of life, and the overthrow of slavery the principal object to be desired. He did a herculean work, and his name will be remembered forever with gratitude by all lovers of freedom. But when the one cause for which he had labored had triumphed, there was nothing of recourse left for him, and the last years of his life were tinged with disquiet, and his end sadened by disappointment.

Wendell Phillips, with all his gifts of birth, of intellect, of moral excellence and broad culture, was not proof against the paralyzing influence of specialism. His long devotion to the promulgation of the principles of anti-slavery, and his life-long work as an anti-slavery agitator so absorbed him, that when the end came and the curse was removed, his work was done, and from that day the fire was gone out from his eye, and his tongue lost its caustic sting. While his life illustrates the point of the subject, we will render all honor to him and feel that such a work was enough for one life, and the knowledge of such results reward enough for a sacrifice of all other ends or desires.

Benjamin Franklin illustrates the better class of minds. Born in poverty, educated in a printing office, possessed of a mind of such power that it might scorn the dull, dreary tread-mill of study, and grasp almost by intuition the facts of science and thoughts of literature for which ordinary minds must delve and struggle, he took upon himself the drudgery of study, set his mind to the labor of thought, and disciplined his faculties into training for the great and varied work of his after life. Instead of limiting himself to the routine of newspaper literature, he became successively the scientist, the statesman and the philosopher, standing a peer among his fellow-countrymen, and challenging the respect and admiration of the world.

To rightly weigh the influence of culture, we must consider the influence of mind upon form and action. It is said in Plato's laws that "spirit precedes the form," and there is given an extended and acute logical argument to demonstrate it. To-day it is quite generally admitted in a little different form. The idea must precede the word, the conception must precede the execution; the hand can go no faster than the mind thinks, and the performance cannot exceed the conception of the intellect. A person color-blind cannot become a scenic artist, nor can a mind without perception of form and proportion make a sculptor. The ideally perfectly balanced man would have all his faculties and powers equally developed, no one showing any predominance, and would possess no controlling taste or talent to decide his course in life. Such a man in this active, specialized life, is fit only for a jack-of-all-trades, and very likely good at none.

While culture calls for the best development of all the faculties, it does not demand that all faculties in all minds shall be alike in strength and persistence. Nature does not make all minds equal or alike in thought or action. This would blot out individuality. It would make a James Watt, or a Stephenson, or a Washington impossible, and the world would settle down into a stagnant mediocrity, or fritter away its power in perpetual strife and disorder, with the shadow of more than the dark ages hovering over it.

The age demands marked ability. Success demands the perfection of one set of talents. The shortness of life makes it impossible to cultivate more than this for practical use; but it is not necessary that all others be neglected until only an automatic monstrosity is the result. Culture demands that all faculties be used, that all tastes and talents be trained, that a well-balanced course of studies be pursued; but it does not demand that one possessing a strong literary taste shall become a carpenter, or that a born philosopher must become a merchant, simply to develop a comparatively weak talent to make the individual well-balanced. This would be following culture for culture's sake to a degree beyond æstheticism. The leading talent must be the controlling force in life. The best life is so governed. The other talents must be disciplined to act as steadying powers to keep the mind on the track; must act as the side-guys of the suspension bridge, keeping it from being swayed by winds and torn from its towers, a complete wreck. Specialism is in itself so contracting that it needs the elevating, exalting, enlarging influence of other ideas to elevate it, and the power of other faculties to keep it within its true limits, and prevent its swaying the mind from the strong towers of common-sense.

The world's progress has been a constant witness to the influence of culture. The advance of the world, morally, socially or materially, has not been of even growth, but of uneven, intermittent strides, and each period of advance in mechanical skill has been the result of a time of mental activity and growth. On the other hand, when the minds of a people grew sluggish, or their thoughts diverted to selfish indulgence, or solely to the art of war, their hands forgot their cunning and their fingers lost their skill. After the Augustinian age, Roman Europe declined. It degenerated under despotism, and arts and sciences declined until, during the fifteenth century, intellectual paralysis prevailed; all arts, save the art of war, became lost arts, and Greece alone saved to the world what Europe had produced, but without the power to develop it. "Rome originated nothing. She gave to the world no new order of architecture, no new science. She invented nothing; for no important tool in any workshop, for no process in our industrial arts, are we indebted to Rome." Charlemagne, the prosperous ruler of the ninth century, divided his kingdom among his sons. Intellectual activity gave place to bodily comfort and appetite, and immediately material interests declined.

From the time of Solomon until the present, intellectual activity and virtue have proven the foundation, support and crown of art, science and material welfare. It is also true of individuals as of nations. No man of the past or present has risen to true eminence in any of the professions who was not both educated and cultured. The surgeon's skill with the scalpel is dependent upon and exceeds not the fineness of the perception of the mind, and the excavator, chisel and drill, in the hands of the dentist, can go only where the mind conceives and the thought directs. The hand intellectualizes the body. "The hand is the symbol of man's power, for while the head wears the crown the hand holds the sceptre. It grasps all instruments for our welfare, from the pen to the plowshare."

No longer can one mind conceive the idea and the unquestioning slave execute the work, but now every artisan must understand the theory of his handicraft. Professional culture is composite—mental and manual. The latter alone makes the unreflecting copyist; the first alone the impotent dude.

In Massachusetts the maxim was early adopted, and has been continually enforced in every period of her history, that "the more cultivated a man's intellect is, the more productive is his labor and the better his life;" and so long as the mind conceives before the hand executes, this must be true. It matters not where the education and the culture is obtained, in college or out, from private teacher or by one's own efforts and application. If it has produced the result of a well-informed and disciplined mind, the work is accomplished. The same is true of professional knowledge. It matters not from whence it comes if it is possessed. It is more easily gotten, and more surely, if the favorable surrounding circumstances of the college and professional schools are sought and improved. But if the obstacles be overcome and the results obtained without these helps, all honor to the victor. In the present, when educational institutions are at everyone's hand, it is poor economy to take the longer and harder route of self-education. Few can do it. Associate education has great advantages. It affords opportunity for interchange of thought. It rubs a man against his fellows, and the friction of emulation brightens faculties and develops excellencies that, without it, would lie dormant through life. It stimulates to good works and furnishes a large share of the discipline of mind and body, which is the essential principle of culture. No one can know the extent or fullness of his own knowledge until it is tried by comparison with others.

This discussion has brought afresh to our attention the fact, which no doubt appeared self-evident from the first, that, added to natural talents, education discipline and culture are the elements of proficiency in every calling, and of success among polite people. The wide diffusion of knowledge, the keener observation of the masses, cause the demands on professional skill to be more exacting and the *ex cathedra* of the doctor to be less readily accepted.

To meet these demands a higher and broader standard of education must be raised, a more thorough preliminary preparation, and a more thorough course of instruction be demanded, and a higher order of talent be encouraged to enter the practice of our profession. Let not the possession of a degree nor the acquirement of a successful practice end intellectual effort, but keep close company with the books, sustain the professional associations, the literary clubs and the scientific societies, and thus keep bright the intellectual faculties, and keep moth, mildew and rust from corrupting and corroding the mental powers.

Culture is the handmaid of art. Keep the hand well trained, for by the hand culture renders her best and greatest service. Bacon's aphorism is expressive and true: "Education is the culture of a legitimate familiarity betwixt the mind and things."

